# Potential Genetic Intersections Between ADHD and Alzheimer’s Disease: A Systematic Review

**DOI:** 10.3390/neurosci6040097

**Published:** 2025-10-01

**Authors:** Riccardo Borgonovo, Lisa M. Nespoli, Martino Ceroni, Lisa M. Arnaud, Lucia Morellini, Marianna Lissi, Leonardo Sacco

**Affiliations:** 1 Department of Neurology, Neuropsychology and Speech Therapy Unit, Neurocenter of Southern Switzerland, Ente Ospedaliero Cantonale, 6900 Lugano, Switzerland; lisamaria.nespoli@eoc.ch (L.M.N.); martino.ceroni@eoc.ch (M.C.); lisamarta.arnaud@eoc.ch (L.M.A.); lucia.morellini@eoc.ch (L.M.); marianna.lissi@eoc.ch (M.L.); leonardo.sacco@eoc.ch (L.S.); 2 Faculty of Biomedical Sciences, Università della Svizzera Italiana, 6900 Lugano, Switzerland

**Keywords:** ADHD, genetic, polygenic risk, neurodegeneration, neurodevelopmental disorders, cognitive decline, molecular pathway

## Abstract

Background: attention-deficit/hyperactivity disorder (ADHD) and Alzheimer’s disease (AD) are distinct neurological conditions that may share genetic and molecular underpinnings. ADHD, a neurodevelopmental disorder, affects approximately 5% of children and 3% of adults globally, while AD, a neurodegenerative disorder, is the leading cause of dementia in older adults. Emerging evidence suggests potential overlapping contributors, including pathways related to synaptic plasticity, neuroinflammation, and oxidative stress. Methods: this systematic review investigated potential genetic predispositions linking Attention-Deficit/Hyperactivity Disorder (ADHD) and Alzheimer’s Disease (AD). Following PRISMA guidelines, a search was conducted in Web of Science, Embase, PsycINFO, and PubMed using keywords related to ADHD, AD, and genetic factors. Studies included were original human studies utilizing genetic analyses and ADHD polygenic risk scores (PRS), with AD confirmed using established diagnostic criteria. Exclusion criteria comprised non-original studies, animal research, and articles not addressing genetic links between ADHD and AD. Screening was conducted with Rayyan software (version 1.4.3), assessing relevance based on titles, abstracts, and full texts. Results:. The search identified 1450 records, of which 1092 were screened after duplicates were removed. Following exclusions, two studies met inclusion criteria. One study analyzed ADHD-PRS in 212 cognitively unimpaired older adults using amyloid-beta (Aβ) PET imaging and tau biomarkers. The findings revealed that ADHD-PRS was associated with progressive cognitive decline, increased tau pathology, and frontoparietal atrophy in Aβ-positive individuals, suggesting that ADHD genetic liability may exacerbate AD pathology. Another study assessed ADHD-PRS in a cohort of 10,645 Swedish twins, examining its association with 16 somatic conditions. The results showed modest risk increases for cardiometabolic, autoimmune, and neurological conditions, with mediation effects through BMI, education, tobacco use, and alcohol misuse, but no direct link between ADHD-PRS and dementia. Discussion and conclusions: this review highlights preliminary but conflicting evidence for a genetic intersection between ADHD and AD. One study suggests that ADHD genetic liability may exacerbate AD-related pathology in Aβ-positive individuals, whereas another large registry-based study finds no direct link to dementia, with associations largely mediated by lifestyle factors. The potential ADHD–AD relationship is likely complex and context-dependent, influenced by biomarker status and environmental confounders. Longitudinal studies integrating genetics, biomarkers, and detailed lifestyle data are needed to clarify this relationship.

## 1. Introduction

Attention-deficit/hyperactivity disorder (ADHD) is a common neurodevelopmental disorder affecting approximately 5% of children and 3% of adults globally [1,2]. While ADHD symptoms, including inattention, hyperactivity, and impulsivity, often persist into adulthood, they frequently manifest differently, with inattention becoming more prominent [3]. The disorder has a strong genetic basis, with heritability estimates around 74%, but environmental factors, such as prenatal alcohol and nicotine exposure, also contribute to its development [4]. Neurobiological studies have identified structural and functional brain differences in individuals with ADHD, particularly in regions related to attention, executive function, and impulse control, including the prefrontal cortex and basal ganglia [5].

ADHD significantly affects quality of life across various domains, including education, work, relationships, and daily functioning. It is frequently accompanied by comorbidities such as mood and anxiety disorders, substance abuse, sleep disturbances, and obesity [6]. In older adults, ADHD is associated with depression and anxiety, which further impair psychosocial functioning and quality of life [7]. Additionally, ADHD is linked to cognitive deficits in attention, memory, inhibition, and executive functioning, which can overlap with symptoms observed in neurodegenerative conditions like Alzheimer’s disease (AD) [8].

AD, a neurodegenerative disorder, is characterized by the progressive accumulation of amyloid-beta (Aβ) plaques and tau tangles, leading to neuronal loss and cognitive decline [9]. Advances in diagnostic methods have shifted AD diagnosis from purely clinical assessments to clinical-biological models incorporating biomarkers such as Aβ and phosphorylated tau in cerebrospinal fluid (CSF) and neuroimaging techniques [10,11]. The ATN (amyloid, tau, neurodegeneration) framework has further refined AD classification [12], though its clinical implementation remains challenging [13].

Recent studies have begun exploring potential genetic and biological overlaps between ADHD and AD, suggesting shared risk factors and molecular pathways. For instance, longitudinal data on ADHD and its progression to AD are sparse but critical for understanding the continuum between these conditions [14]. Emerging genetic studies, such as those identifying polygenic risk scores (PRS), indicate that ADHD genetic liability may influence neurodegenerative processes, particularly in the presence of Aβ and tau biomarkers [15]. Genetic analyses have identified several genes implicated in both ADHD and AD, including *FOXP2*, *FOXP1*, *ST3GAL3*, *SEMA6D*, *SORCS2*, *SORCS3*, *SNAP25*, *DUSP6*, *MEF2C*, and *APOE ε4*, highlighting their roles in synaptic plasticity, neuronal viability, and cognitive processes [14,16,17]. These findings suggest that genetic pleiotropy, where shared genetic variants influence both conditions, may underpin their association. Key pathways include Wnt/mTOR signaling, neuroinflammation, and oxidative stress, all of which contribute to synaptic dysfunction and neurodegeneration [18,19]. 

Understanding these genetic and biological overlaps provides a foundation for exploring how ADHD predispositions may influence late-life cognitive decline and AD risk. It also emphasizes the need for longitudinal studies, advanced genetic profiling, and biomarker integration to elucidate the ADHD-AD continuum and inform prevention and intervention strategies.

## 2. Materials and Methods

### 2.1. Search Strategy, Study Selection and Data Extraction

The aim of this systematic review was to systematically collect and synthesize evidence on the potential genetic predispositions linking ADHD and AD, with particular focus on polygenic risk scores (PRS) and overlapping molecular pathways. The present systematic review was conducted according to the Preferred Reporting Items for Systematic Reviews and Meta-Analyses (PRISMA) [20]. The method is currently available in the Open Science Framework (OSF, https://osf.io/hn7mc/, accessed on 21 June 2025). A search in the main electronic databases (Web of Science, Embase, Psychinfo, and PubMed) was performed using the keywords (“ADHD” OR “attention deficit disorder” OR “Hyperactive Disorder” OR “Hyperkinetic Disorder” OR “Attention Disorder”) AND (“dementia” OR “Alzheimer” OR “Alzheimers” OR “cognitive impairment”) AND (“gene” OR “genes” OR “genetic” OR “genetics” OR “Polygenic” OR “mutation” OR “mutations” OR “polymorphism” OR “SNP”). The systematic review protocol has been registered in the PROSPERO database (PROSPERO ID CRD420251077681. Available from https://www.crd.york.ac.uk/PROSPERO/view/CRD420251077681, accessed on 21 June 2025).

The study was conducted from October 2023 to June 2025. After an initial selection, the inclusion criteria were deliberately strict and required: (i) original human studies only; (ii) genetic analyses conducted exclusively through sequencing methods (excluding karyotyping, CGH array, or other indirect approaches); (iii) studies reporting both a remote pathological history of ADHD and a subsequent diagnosis of AD; and (iv) confirmation of AD diagnosis through cerebrospinal fluid (CSF) biomarkers. However, this strategy did not yield any eligible studies.

Consequently, the criteria were subsequently revised and broadened to better reflect the available literature. The final inclusion criteria applied were: (i) studies involving participants diagnosed with adult ADHD or using ADHD polygenic risk scores (PRS) as a proxy for genetic liability; (ii) studies explicitly addressing both ADHD and AD simultaneously; (iii) articles written in English; and (iv) studies conducted in human populations.

The exclusion criteria remained unchanged and included: reviews, meta-analyses, letters to the editor, commentaries, case reports, pooled analyses, and study protocols; animal studies; articles not written in English; and studies not addressing potential genetic links between ADHD and AD or cognitive decline.

The screening process was carried out using Rayyan software. Titles and abstracts were first screened for relevance, followed by full-text evaluation of the methodology and results to determine final eligibility.

### 2.2. Polygenic Risk Score Methodology

Polygenic risk scores (PRS) summarize genomic liability by weighting risk alleles using genome-wide association studies (GWAS) effect sizes (e.g., PGC ADHD GWAS). ADHD-PRS derive from large ADHD GWAS [16] and provide an aggregate index of ADHD genetic liability. In AD-focused analyses, ADHD-PRS can be related to cognition and AD biomarkers (Aβ PET, CSF p-tau) and neuroimaging measures.

### 2.3. AI Usage Statement

The authors declare the use of AI-based tools (Academic Assistant Pro 5) in the study methodology, specifically for language editing and figure generation. These tools were employed to support the refinement of the manuscript’s syntax, ensuring clarity and consistency in the text. Additionally, AI was used to assist in the creation of figures, which were integral to the data visualization in the manuscript. However, the scientific content, data interpretation, and conclusions remain the exclusive responsibility of the authors.

## 3. Results

The search identified a total of 1450 records from the following databases: Web of Science (n = 124), Embase (n = 1105), PsychInfo (n = 77), and PubMed (n = 144). After the automated removal of duplicate records (n = 358), 1092 records were screened.

During the screening process, 1084 records were excluded for the following reasons: non-original studies (n = 386), incongruent topics (n = 358), not discussing AD genetics (n = 162), non-human studies (n = 88), not discussing ADHD genetics (n = 75), background articles (n = 19), and articles in foreign languages (n = 4).

**Figure 1:**
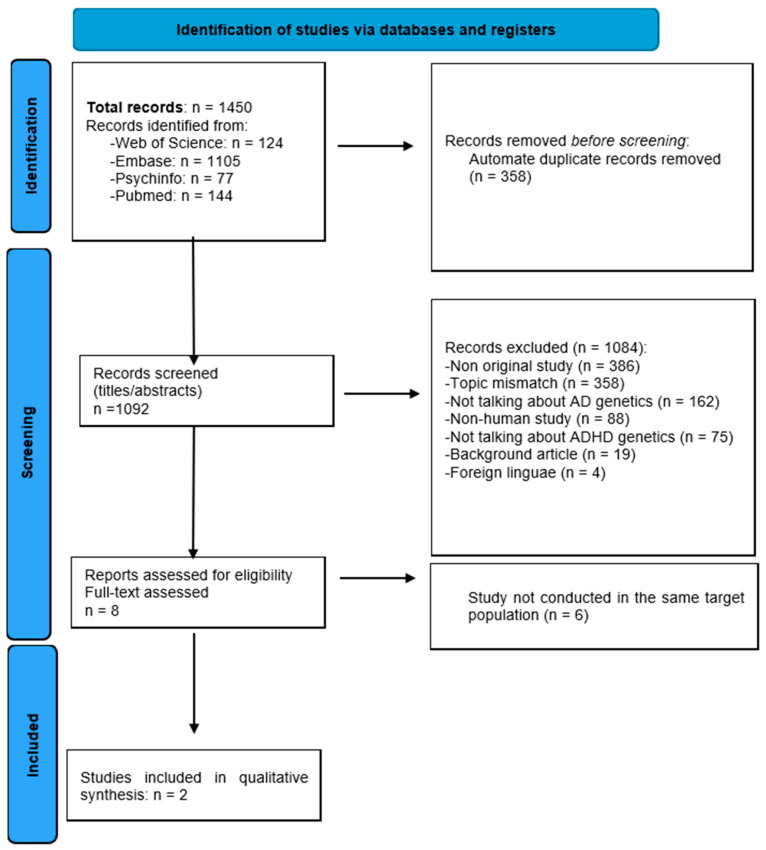
PRISMA 2020 flow diagram summarizing study selection (identification, screening, eligibility, inclusion). Included studies: Leffa et al. 2023 [15] and García-Argibay et al. 2022 [21].

Subsequently, eight reports were assessed for eligibility. Among these, six studies were excluded because they were not conducted on the same population. Finally, two studies met the inclusion criteria and were included in the analysis. The selection process is reported in Figure 1.

Two studies were selected, both evaluating the presence of an ADHD polygenic risk score (PRS) in individuals with dementia. Polygenic risk scores quantify an individual’s genetic predisposition to specific diseases or traits by aggregating the effects of numerous genetic variants across the genome. The studies are reported in Table 1.

Leffa et al. (2023) [15] utilized polygenic risk scores (PRS) to explore the genetic relationship between ADHD and Alzheimer’s disease (AD) pathology in 212 cognitively unimpaired older adults. Their analysis incorporated ADHD-PRS, amyloid-beta (Aβ) PET imaging, and tau biomarkers, revealing a significant association between higher ADHD-PRS and progressive cognitive decline over six years. Memory function was particularly impacted, with the decline being more severe in individuals positive for Aβ. Additionally, higher ADHD-PRS correlated with increased cerebrospinal fluid (CSF) phosphorylated tau (p-tau181) levels and greater brain atrophy in frontoparietal regions, specifically in Aβ-positive individuals. These findings suggest that genetic liability for ADHD not only predisposes individuals to cognitive decline but also exacerbates key pathological processes in AD, such as tauopathy and neurodegeneration, particularly in the presence of Aβ pathology [15].

Garcia-Argibay et al. (2022) [21] examined the impact of ADHD polygenic risk scores (PRS) on mid-to-late life somatic health conditions in a population-based cohort of 10,645 Swedish twins. The study assessed associations between ADHD-PRS and 16 health conditions, including cardiometabolic, autoimmune/inflammatory, and neurological outcomes, using both register-based and self-reported data.

Significant associations were found between higher ADHD-PRS and increased risks for cardiometabolic conditions (e.g., heart failure, cerebrovascular disease, and obesity), autoimmune conditions (e.g., type 1 diabetes and rheumatoid arthritis), and migraines. The study identified mediation effects through modifiable life-course factors, such as education level, body mass index (BMI), tobacco use, and alcohol misuse, particularly for cardiometabolic outcomes. However, no direct association was observed between ADHD-PRS and dementia, contrasting with other studies linking ADHD genetic liability to cognitive decline. These findings suggest that ADHD genetic risk contributes modestly to somatic health problems via modifiable behavioral and lifestyle pathways.

Leffa et al. (2023) and Garcia-Argibay et al. (2022) [15,21] collectively provide critical insights into the downstream effects of ADHD genetic liability. While Leffa et al. [15] link ADHD-PRS to Alzheimer’s disease pathology and cognitive decline, Garcia-Argibay et al. [21] highlight its broader influence on mid-to-late life somatic health conditions, mediated by modifiable factors. Together, these studies underscore the need for tailored interventions to mitigate the risks associated with ADHD genetic predisposition, addressing both cognitive and somatic health trajectories.

## 4. Discussion

This systematic review examines the nascent evidence for shared genetic and molecular bases between ADHD and AD, based on two recent studies utilizing polygenic risk scores. Leffa et al. (2023) [15] investigated ADHD polygenic risk scores (PRS) in 212 cognitively unimpaired older adults with Aβ PET and tau biomarkers. Higher ADHD-PRS predicted six-year cognitive decline, particularly in memory, and was associated with increased CSF p-tau181 and frontoparietal atrophy, but only in Aβ-positive individuals. These findings suggest that ADHD genetic liability may exacerbate AD-related pathology in the presence of amyloid.

Garcia-Argibay et al. (2022) [21], analyzing 10,645 Swedish twins, found that higher ADHD-PRS modestly increased risks for cardiometabolic, autoimmune, and neurological conditions, with effects largely mediated by BMI, education, tobacco, and alcohol. Crucially, no direct association with dementia was observed.

Together, these studies illustrate the heterogeneity of current evidence: a biomarker-based cohort showed a direct ADHD-PRS link to AD pathology, while a large registry study found null dementia associations, emphasizing the role of modifiable factors. This contrast suggests the ADHD–AD relationship is likely context-dependent, subtype-specific, and shaped by study design, requiring integrative research to resolve discrepancies.

### 4.1. Lack of Longitudinal Studies 

Despite growing evidence of shared genetic markers, there is a notable lack of longitudinal studies assessing whether individuals with ADHD are more likely to develop AD later in life, particularly among those genetically predisposed to neurodegeneration. Advances in diagnostic criteria, genetic technologies, and the identification of overlapping genetic markers between ADHD and AD have emerged only recently. These developments highlight the critical need for future longitudinal and retrospective studies to elucidate the continuum between these conditions [14].

### 4.2. Epidemiological Evidence of Genetic Correlation

Emerging epidemiological studies provide compelling evidence of a genetic correlation between ADHD and AD. For instance, Leffa et al. (2022) [15] reported that individuals with higher polygenic risk scores (PRS) for ADHD experienced more rapid memory decline, particularly in the presence of AD biomarkers such as amyloid-beta (Aβ) and phosphorylated tau (p-tau181, emphasizing the shared genetic liability and structural brain changes common to both disorders.

### 4.3. Genetic Contributors

Framing note (important): While the two included PRS studies suggest an aggregate genetic overlap between ADHD and AD, they do not identify specific causal genes. Accordingly, the following list of candidate genes is drawn from the broader literature (e.g., GWAS, candidate-gene and mechanistic studies) to hypothesize potential biological drivers of the PRS signals. These links are hypothesis-generating and should be interpreted as speculative, not as direct results of the included PRS analyses.

The following genes have been implicated in both ADHD and AD, with their roles derived from genome-wide association studies (GWAS), SNP analyses, and targeted genetic investigations.

*FOXP2*: the *FOXP2* gene encodes a transcription factor involved in synapse formation and learning, highlighting its critical role in neural connectivity. In ADHD and Alzheimer’s disease (AD), neural connectivity disruptions are shared pathological features. *FOXP2* regulates several downstream genes crucial for synaptic development and plasticity, implicating its dysfunction in cognitive and behavioral abnormalities [16] Studies also suggest that alterations in *FOXP2* expression impact language acquisition and cognitive processing, which are affected in both ADHD and the early stages of AD [22].

*FOXP1*: functionally related to *FOXP2*, *FOXP1* regulates neurodevelopmental pathways, including axonal growth and synaptic connectivity. Mutations in *FOXP1* are associated with neurodevelopmental disorders and cognitive impairments, conditions that share overlapping features with AD [16]. *FOXP1* deficiency disrupts the expression of target genes critical for neuronal differentiation and survival, potentially contributing to the neurodegeneration observed in AD [23].

*ST3GAL3*: this gene encodes a sialyltransferase enzyme involved in glycoprotein biosynthesis, a process essential for maintaining synaptic integrity and plasticity. Mutations in *ST3GAL3* impair glycosylation pathways, leading to deficits in neuronal communication and cognitive function. These mutations have been implicated in intellectual disabilities and neurodevelopmental disorders, suggesting a role in the cognitive decline shared by ADHD and AD [14]. Furthermore, glycosylation anomalies have been linked to amyloid precursor protein (APP) processing, a hallmark of AD pathology [24].

*SEMA6D*: *SEMA6D* encodes a semaphorin protein essential for neuronal development and axon guidance. Variants in this gene disrupt axonal patterning and synaptic connectivity, processes critical for maintaining cognitive function. Dysregulation of *SEMA6D* has been associated with ADHD and AD, where axonal misguidance and impaired neural circuitry contribute to disease progression [16,25].

*SORCS2* and *SORCS3*: these genes encode sorting receptors that regulate neuronal viability, synaptic plasticity, and APP processing. *SORCS2* interacts with APP to influence its cleavage into amyloid-beta, a protein implicated in AD pathology. Variants in *SORCS3* disrupt synaptic transmission and plasticity, processes that underlie cognitive deficits in ADHD and AD. Emerging evidence highlights the role of these receptors in neuroprotection and their potential as therapeutic targets in AD [26].

*SNAP25*: a core component of the synaptic vesicle fusion machinery, *SNAP25* is essential for neurotransmitter release. Polymorphisms in *SNAP25* have been linked to altered dopamine signaling in ADHD, while reduced protein levels are observed in AD, contributing to synaptic dysfunction. Dysregulation of *SNAP25* affects synaptic vesicle recycling, a process essential for maintaining neuronal communication and plasticity [27].

*DUSP6*: dual specificity phosphatase 6 (*DUSP6*) regulates dopamine homeostasis and mitogen-activated protein kinase (MAPK) pathways. These pathways are critical in modulating synaptic plasticity and cognitive processes. Dysregulation of *DUSP6* has been implicated in ADHD due to its effects on dopamine signaling, while in AD, altered MAPK signaling contributes to neurodegeneration [16,28].

*MEF2C*: the *MEF2C* gene encodes a transcription factor that regulates neuronal activity and plasticity. Disruptions in *MEF2C* expression are associated with severe cognitive impairments in ADHD and neurodegeneration in AD. *MEF2C* is involved in synaptic pruning and neuroinflammatory regulation, suggesting that its dysfunction may exacerbate pathological processes in AD, such as synaptic loss and amyloid deposition [16,29].

*APOE ε4*: the *APOE ε4* allele is a well-established genetic risk factor for AD, influencing amyloid-beta aggregation and clearance. It is also linked to cognitive dysfunction in ADHD, suggesting a shared genetic vulnerability. The ε4 variant exacerbates synaptic dysfunction and neuroinflammation, processes common to both conditions [17,30].

*BDNF*: brain-derived neurotrophic factor (*BDNF*) is critical for neuronal survival, growth, and plasticity. Polymorphisms in the *BDNF* gene, such as the Val66Met variant, impair *BDNF* trafficking and secretion, contributing to synaptic and cognitive deficits in ADHD and AD [31,32]. Reduced *BDNF* levels in AD patients correlate with disease severity, highlighting its therapeutic potential.

*APP*: variants in the *APP* gene, central to AD pathology, are also associated with cognitive deficits in ADHD, particularly during childhood [17]. APP processing anomalies lead to amyloid-beta accumulation, a key driver of AD, and may contribute to synaptic dysfunction observed in ADHD [33].

*NUAK1*: *NUAK1* is involved in cortical amyloid-beta regulation and neuronal plasticity. Its role in synaptic remodeling is critical for attention regulation in ADHD and for counteracting cognitive decline in AD [34]. Dysregulated *NUAK1* activity exacerbates amyloid toxicity and synaptic loss, key features of AD progression [35].

*KIF21B*: a kinesin family member, *KIF21B* is essential for intracellular transport and neuronal homeostasis. Dysfunctional *KIF21B* impacts axonal transport mechanisms, leading to neurodegeneration in AD and contributing to cognitive impairments in ADHD [36]. Impaired transport of organelles and synaptic vesicles underscores its role in both developmental and degenerative processes. 

The main gene involved in ADHD and AD expression are reported in Table 2. 

### 4.4. Molecular Pathways Involved 

**Figure 2:**
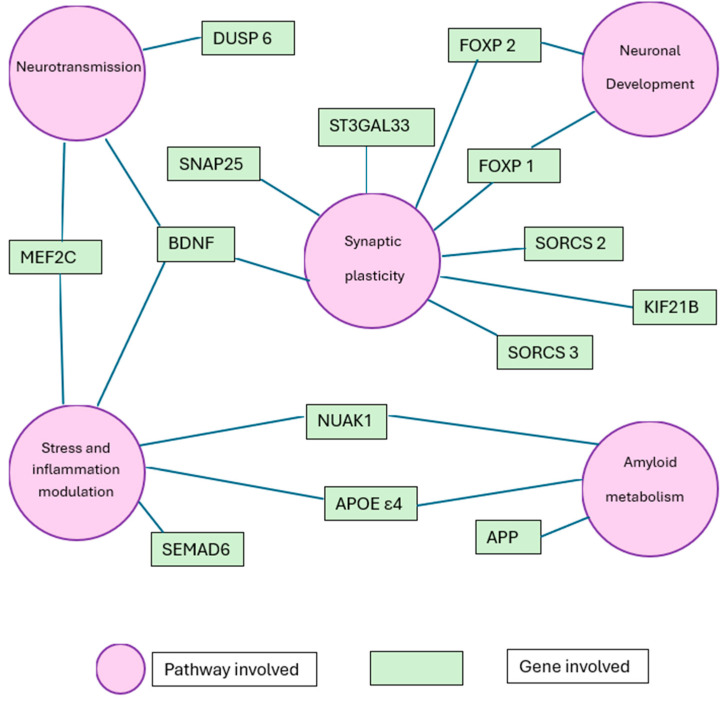
Conceptual network map showing shared genes and biological pathways between Attention-Deficit/Hyperactivity Disorder (ADHD) and Alzheimer’s Disease (AD).

Recent studies have illuminated shared molecular pathways between Attention-Deficit/Hyperactivity Disorder (ADHD) and Alzheimer’s Disease (AD), suggesting common pathogenic mechanisms. In Figure 2 is reported a network map showing the relationship between these genes and cellular pathway involved.

### 4.5. Functional Roles and Contradictions in Gene–Pathway Interactions

While the identification of shared genes such as *APOE ε4*, *SNAP25*, and *FOXP2* underscores the potential overlap between ADHD and AD, their functional contributions are complex and often context-dependent.

Pathway-level analyses illustrate that these genes converge on critical neurobiological processes, including synaptic plasticity, axonal guidance, and neuroinflammation. For example, *FOXP2* and *FOXP1* regulate transcriptional programs essential for synaptic development, influencing pathways such as Wnt/β-catenin and MAPK signaling, which are dysregulated in both ADHD and AD. Experimental evidence highlights that *FOXP2* disruption alters dendritic spine density and learning processes, while in AD models, its dysregulation exacerbates cognitive decline and hippocampal dysfunction. Likewise, *SORCS2* and *SORCS3* intersect with amyloid precursor protein (APP) processing, a key mechanism in AD, while also modulating synaptic plasticity relevant to attentional regulation in ADHD [16].

Contradictions emerge when the same gene exerts divergent effects across conditions. For instance, while reduced *BDNF* signaling is consistently observed in AD and correlates with disease severity, certain ADHD cohorts have shown enhanced *BDNF* expression as a compensatory response to altered dopaminergic circuits. Similarly, *DUSP6* dysregulation increases vulnerability to ADHD through dopamine imbalance but has been reported to mitigate MAPK hyperactivation in AD, suggesting potential protective effects in the neurodegenerative context [14,16].

Taken together, these examples underscore the importance of moving beyond gene identification to critically assess functional directionality. Genes implicated in both ADHD and AD may not uniformly act as risk factors; rather, their effects appear to be shaped by developmental timing, cellular context, and environmental exposures. This highlights the need for experimental and longitudinal studies integrating molecular, behavioral, and clinical data to disentangle causal contributions from compensatory or protective mechanisms. 

### 4.6. Wnt/mTOR Signaling Pathway 

The Wnt/β-catenin signaling pathway is essential for neuronal survival and neurogenesis. Impairments in this pathway are linked to increased amyloid-beta levels and decreased β-catenin, both implicated in AD pathogenesis [37]. Additionally, hyperactivation of the mTOR pathway promotes tau hyperphosphorylation and amyloid-beta aggregation, key processes in AD [18]. Interestingly, methylphenidate, a common ADHD treatment, has been shown to modulate Wnt/mTOR pathway activity, indicating a potential therapeutic link between the two conditions [18].

### 4.7. Neuroinflammation and Oxidative Stress 

Chronic neuroinflammation and oxidative stress are prevalent in both ADHD and AD. Mitochondrial dysfunction contributes to these processes, leading to neuronal damage in both disorders [18,19]. Persistent neuroinflammation can accelerate neurodegeneration, suggesting a shared mechanism of neuronal injury.

### 4.8. Glycosylation

Aberrant glycosylation, mediated by enzymes such as *ST3GAL3*, affects glycoprotein biosynthesis, altering synaptic connectivity. These changes have been observed in both ADHD and AD, indicating a possible shared pathogenic mechanism [14].

### 4.9. Controversy

This evidence points to a potential genetic link between Attention-Deficit/Hyperactivity Disorder (ADHD) and Alzheimer’s Disease (AD), but the association remains contentious due to several confounding factors. Pagoni et al. [38] conducted a study to explore this possible relationship further. Utilizing a two-sample Mendelian randomization approach, the research investigated the bidirectional causal association between genetic liability to ADHD and AD. Genetic instruments were derived from large-scale GWAS datasets to estimate causal effects independently of confounders such as educational attainment and IQ. Sensitivity analyses were performed to enhance the robustness of the finding.

Despite observational studies hypothesizing a link between ADHD and increased AD risk, Pagoni et al. (2022) [38] found no strong evidence of a causal relationship. Their findings suggest that previously reported associations may be influenced by shared environmental and familial factors, including socioeconomic status and comorbid conditions. Additionally, the heterogeneity of ADHD phenotypes and reliance on aggregated data might obscure differential risks associated with specific ADHD subtypes [38].

An additional limitation of this review is the very small number of eligible studies (n = 2), which substantially reduces the generalizability of our conclusions, increases the risk of type II errors.

### 4.10. Limitations: Role of Modifiable Risk Factors

While genetic correlations between ADHD and AD are increasingly reported, the potential role of confounding variables must be acknowledged. Lifestyle factors (e.g., diet, physical activity, smoking), comorbid psychiatric conditions such as depression, and medication effects may significantly influence both cognitive decline and the expression of ADHD-related traits in later life. Garcia-Argibay et al. (2022) [21] reported that the association between ADHD and AD was mediated primarily through body mass index (BMI) and educational attainment, with no direct causal relationship observed, thereby underscoring the relevance of environmental and socioeconomic interactions.

Furthermore, Mendelian randomization (MR) studies have provided valuable tools to disentangle correlation from causation. For instance, Pagoni et al. (2022) [38] found no strong evidence supporting a direct genetic liability link between ADHD and AD, suggesting that shared environmental exposures and modifiable risk factors may account for much of the observed association. These findings emphasize the importance of considering non-genetic contributors when interpreting the overlap between ADHD and AD, and highlight the need for integrative research designs that combine genetic, environmental, and lifestyle data. Overall, the combination of small biomarker cohorts and large registry-based outcome cohorts suggests that PRS-ADHD ↔ neurodegenerative outcome associations may only emerge in specific biological contexts (e.g., Aβ+), while at the population level they are attenuated by path-dependent confounders (education, BMI, habits).

### 4.11. Clinical Implications 

The genetic and molecular overlaps between ADHD and AD suggest potential clinical relevance, but current evidence remains preliminary. Rather than advancing speculative therapeutic overlap, these findings should be considered a foundation for early identification and prevention strategies. In pediatric populations, closer monitoring of ADHD subtypes carrying genetic variants linked to neurodegenerative processes, such as *APOE ε4* or *SNAP25*, may be justified, particularly through longitudinal biomarker screening for amyloid-beta and phosphorylated tau. In adults, especially those with vascular or psychiatric comorbidities, ADHD may represent a subgroup at increased risk of accelerated cognitive decline. Early cognitive assessments combined with neuroimaging and cerebrospinal fluid biomarkers could help stratify the risk of AD onset.

Pharmacological treatments commonly prescribed in ADHD, such as dopaminergic agonists and stimulants, should not be regarded as immediate therapeutic options for AD. However, their known action on dopaminergic transmission and on pathways such as Wnt/mTOR and synaptic plasticity makes them biologically relevant candidates for further investigation. These agents should be evaluated systematically in controlled studies, not as symptomatic treatments for dementia, but as potential modulators of shared molecular mechanisms that might influence neurodegenerative trajectories.

Future research must therefore prioritize longitudinal studies across the lifespan, distinguishing pediatric- from adult-onset ADHD, to clarify whether dopaminergic modulation exerts long-term protective or detrimental effects in relation to AD. It is equally important to assess whether early lifestyle and psychosocial interventions, combined with cautious evaluation of ADHD pharmacotherapies, can reduce the likelihood of later-life neurodegeneration [18,39].

### 4.12. Pharmacological Interventions 

Targeting common pathways such as oxidative stress, neuroinflammation, and mTOR dysregulation may yield treatments effective for both ADHD and AD. For instance, modulating the mTOR pathway has shown promise in addressing tau pathology in AD [18]. Additionally, interventions aimed at reducing neuroinflammation could potentially mitigate symptoms in both disorders.

### 4.13. Precision Medicine Approaches 

Leveraging genetic profiles enables the design of individualized treatment plans, enhancing efficacy and minimizing adverse effects. By understanding specific genetic predispositions, clinicians can tailor interventions that address the unique molecular underpinnings of each patient’s condition [18].

### 4.14. Emerging Therapeutic Strategies 

Recent advancements suggest novel approaches that could benefit both ADHD and AD patients: 

Endocannabinoid System Modulation: Cannabinoids have demonstrated therapeutic potential in neurodevelopmental and neurodegenerative disorders, indicating a possible role in treating ADHD and AD by modulating neuromodulatory functions [40].

Autophagy Enhancement: Stimulating autophagy processes may aid in clearing protein aggregates, a common feature in AD pathology, and could have implications for ADHD treatment strategies [41]. 

### 4.15. Future Research Directions 

Advancing our understanding of the potential link between Attention-Deficit/Hyperactivity Disorder (ADHD) and Alzheimer’s Disease (AD) necessitates targeted research efforts. Key areas for future investigation include: 

### 4.16. Development of Detailed ADHD Polygenic Risk Profiles 

Creating comprehensive polygenic risk scores (PRS) for ADHD can help identify individuals at increased risk for AD. By analyzing genetic variants associated with ADHD, researchers can assess the likelihood of developing AD-related cognitive decline. Leffa et al. (2023) [15] demonstrated that higher ADHD-PRS correlates with progressive cognitive decline and AD biomarkers, underscoring the importance of genetic profiling in early risk detection.

### 4.17. Longitudinal Studies Tracking ADHD Populations 

Conducting long-term studies that monitor individuals with ADHD into older age is crucial for evaluating the development of AD. Such research can elucidate the temporal relationship between ADHD and AD onset, providing insights into potential causative links. Current literature indicates a need for extensive longitudinal data to understand this progression fully. 

### 4.18. Retrospective Analyses of ADHD Symptomatology in AD Patients 

Investigating the prevalence and impact of ADHD-related symptoms in individuals with established AD can reveal shared pathophysiological features. Retrospective studies focusing on early-life ADHD symptoms in AD patients may identify commonalities that inform prevention and treatment strategies. Leffa et al. (2023) [15] highlight the significance of such analyses in understanding the ADHD-AD continuum. 

Pursuing these research directions will deepen our comprehension of the potential ADHD-AD connection and guide the development of early interventions for at-risk populations.

## 5. Limitations

Only two studies met the inclusion criteria, precluding any meta-analysis and substantially limiting statistical power. This raises the risk of Type II error and inflates imprecision around effect estimates. Heterogeneity in methodology (biomarker-anchored PRS analyses vs. registry-based somatic outcomes) further complicates direct comparisons. We therefore interpret the direction and magnitude of associations as hypothesis-generating rather than confirmatory. The present study highlights limitations due to the lack of literature investigating a direct genetic correlation between the presence of Attention-Deficit/Hyperactivity Disorder (ADHD) and the development of Alzheimer’s Disease (AD). Only two studies [15,21] suggested that genetic liability for ADHD may contribute to AD pathology (particularly in individuals with existing amyloid pathology, potentially accelerating progression) and that ADHD genetic liability influences several mid-to-late life somatic health conditions (primarily through modifiable risk factors like BMI and lifestyle).

More specifically, the first study by Leffa et al. [15] provides valuable longitudinal evidence on how genetic risk for ADHD may predict cognitive decline and the development of AD-related pathology in cognitively unimpaired older adults, directly addressing our core research focus. The second study by Garcia-Argibay et al. [21], while centered more broadly on somatic health conditions associated with ADHD genetic risk in mid-to-late life, also offers important insights into possible neurodegenerative implications, making it a relevant contribution to our review.

Despite finding only two studies emphasizing a correlation between the presence of ADHD and the development of AD, in the present study we pointed out a variety of factors implicated in the correlation between ADHD and AD. 

The limited number of studies eligible for inclusion in our systematic review is primarily due to the highly specific nature of the research question we addressed, focusing on the genetic correlation between ADHD and the risk of developing Alzheimer’s disease (AD) over the lifespan. Despite an initial pool of numerous records, only two studies met the stringent inclusion criteria that ensured direct relevance to this complex intersection.

The scarcity of eligible literature reflects both the newness and the interdisciplinary nature of this research area, which lies at the intersection of psychiatric genetics and neurodegeneration—a field still in its early stages. Furthermore, many existing studies explore ADHD and Alzheimer’s disease independently or focus on clinical and genetic aspects in isolation, rather than investigating their potential genetic linkage over the lifespan. 

Our strict criteria, designed to maximize the relevance and quality of included evidence, inevitably narrowed the pool of suitable studies but were necessary to maintain the focus of the review.

### Need for Future Research 

Although an increasing body of evidence supports the existence of shared genetic markers, there remains a substantial lack of longitudinal research investigating whether individuals with ADHD are at greater risk of developing Alzheimer’s disease later in life—particularly among those with a genetic predisposition to neurodegeneration. Recent advances in diagnostic frameworks, genetic methodologies, and the identification of overlapping genetic risk factors between ADHD and AD underscore the pressing need for future longitudinal and retrospective studies, which integrate genetics and neuropsychological approaches, aimed at elucidating the potential pathophysiological continuum between these conditions.

## 6. Conclusions

This review identifies a stark contrast in current evidence: one biomarker-rich study suggests that ADHD genetic liability may exacerbate AD-related pathology and cognitive decline in Aβ-positive individuals, whereas a large population study finds no direct genetic link to dementia, with associations mediated by modifiable factors. Any potential ADHD–AD relationship is likely complex, context-dependent, and shaped by biomarker status and environmental confounders. The most pressing need is for longitudinal studies integrating genetics, biomarkers, and detailed lifestyle data to resolve these conflicting findings and to determine clinical utility.

## Figures and Tables

**Table 1:** Description of the studies considered.

Study	Methodology	Population	ADHD Measure	PRS Source	AD Outcome/Biomarkers	Key Findings	Implications
Leffa et al., 2023 [15]	Polygenic risk score (PRS) analysis in 212 cognitively unimpaired older adults using ADHD-PRS, amyloid-beta (Aβ) PET imaging, and tau biomarkers.	212 cognitively unimpaired older adults; included measures of amyloid-beta (Aβ) deposition and tau pathology through PET imaging	ADHD-PRS	PGC ADHD GWAS	Aβ PET, CSF p-tau181, MRI, cognition	ADHD-PRS was associated with progressive cognitive decline, increased tau pathology, and frontoparietal atrophy in Aβ-positive individuals	Suggests that genetic liability for ADHD may contribute to Alzheimer’s disease (AD) pathology, particularly in individuals with existing amyloid pathology, potentially accelerating progression.
García-Argibay et al., 2022 [21]	Polygenic risk score (PRS) analysis in a large twin cohort from Sweden, assessing the association of ADHD-PRS with 16 somatic conditions, including dementia, cardiometabolic, autoimmune, and neurological disorders. Mediation and moderation analyses explored effects of life-course factors like BMI, education, tobacco use, and alcohol misuse.	10,645 Swedish twins born between 1911 and 1958. Outcomes assessed through national health registers, self-report data, and clinical diagnoses. Included conditions like dementia, heart failure, cerebrovascular disease, migraine, and obesity.	ADHD-PRS	PGC ADHD GWAS	Somatic conditions registry (incl. dementia)	ADHD-PRS was associated with modest risk increases for cardiometabolic outcomes (heart failure, cerebrovascular disease, obesity), autoimmune conditions (type 1 diabetes, rheumatoid arthritis), and neurological outcomes (migraine). Mediation effects through BMI, education, tobacco use, and alcohol misuse were significant for many outcomes. Dementia was not directly linked to ADHD-PRS.	Suggests that ADHD genetic liability influences several mid-to-late life somatic health conditions, primarily through modifiable risk factors like BMI and lifestyle. No direct genetic link to AD or dementia was found, contrasting with other studies that implicate ADHD-PRS in cognitive decline.

**Table 2:** Description of the genes involved in ADHD and AD expression.

Gene	Encoded Protein	Function of the Protein	Author
*FOXP2*	Forkhead Box P2	Associated with synapse formation and learning. Highlights neural connectivity as a shared factor in ADHD and AD.	[16,22]
*FOXP1*	Forkhead Box P1	Involved in neurodevelopmental processes. Alterations linked to cognitive impairments and overlapping pathways with AD.	[16,23]
*ST3GAL3*	ST3 Beta-Galactoside Alpha-2,3-Sialyltransferase 3	Plays a role in glycoprotein biosynthesis and synaptic plasticity. Mutations associated with cognitive deficits in both conditions.	[14,24]
*SEMA6D*	Semaphorin 6D	Critical for neuronal development and axon guidance. Variants implicated in ADHD and AD pathologies	[16,25]
*SORCS2*	Sortilin-Related VPS10 Domain	Encodes a receptor essential for neuronal viability. Linked to amyloid precursor protein (APP) processing	[16]
*SORCS3*	Sortilin-Related VPS10 Domain	Encodes a receptor critical for neuronal plasticity and cognitive function.	[16]
*SNAP25*	Synaptosomal-Associated Protein 25	Crucial for synaptic vesicle fusion and neurotransmitter release. Linked to ADHD polymorphisms and reduced protein levels in AD	[27]
*DUSP6*	Dual Specificity Phosphatase 6	Regulator of dopamine homeostasis. Influences neurotransmitter pathways relevant to ADHD and AD.	[16,28]
*MEF2C*	Myocyte Enhancer Factor 2C	Regulates neuronal activity. Links severe cognitive impairments in ADHD and neurodegeneration in AD.	[16]
*APOE ε4*	Apolipoprotein E ε4	A well-established AD risk factor. Linked to cognitive dysfunction in ADHD, suggesting shared genetic vulnerability.	[30]
*BDNF*	Brain-Derived Neurotrophic Factor	Polymorphisms affect neuronal survival and plasticity, contributing to vulnerabilities in ADHD and AD.	[31,32]
*APP*	Amyloid Precursor Protein	Variants central to AD pathogenesis. Associated with cognitive deficits in ADHD, particularly in childhood	[30]
*NUAK1*	AMPK-Related Protein Kinase	Involved in cortical amyloid-beta protein regulation and neuronal plasticity. Crucial for attention regulation in ADHD and cognitive decline in AD.	[34,35]
*KIF21B*	Kinesin Family Member 21B	Associated with neurodegenerative disorders and neuronal transport. Dysregulation linked to Alzheimer’s and ADHD.	[36]

## Data Availability

Data are contained within the article.
